# Relationships Between Weekly Dynamic Stress Load Volume and Match-Play External and Internal Load: Half-Specific and Full-Competition Analyses in Professional Soccer Players

**DOI:** 10.3390/s26082496

**Published:** 2026-04-17

**Authors:** Nikolaos E. Koundourakis, Nikolaos Androulakis, Minas Panagiotis Ispirlidis, Dimitra Sifaki-Pistolla, Michalis Mitrotasios, Adam L. Owen

**Affiliations:** 1 Faculty of Sports Science and Physical Education, Metropolitan College, Campus Crete, 71202 Heraklion, Greece; 2 Department of Clinical Chemistry/Biochemistry, School of Medicine, University of Crete, Voutes, 71003 Heraklion, Greece; 3 Sports Science Department, OFI Crete FC, 71303 Heraklion, Greece; 4 Hematology Laboratory, University Hospital of Heraklion, 71500 Heraklion, Greece; nikandgr@gmail.com; 5 Department of Physical Education and Sports Science, School of Physical Education, Sports and Occupational Therapy Science, Democritus University of Thrace, 69100 Komotini, Greece; 6 Faculty of Psychology, Metropolitan College, Campus Crete, 71202 Heraklion, Greece; dsifaki@mitropolitiko.edu.gr; 7 School of Physical Education & Sport Science, National & Kapodistrian University of Athens, 10558 Athens, Greece; micmit@phed.uoa.gr; 8 Research and Innovation Center on Sport (CRIS), Claude Bernard University Lyon 1, 69100 Villeurbanne, France; adamowen@outlook.com

**Keywords:** dynamic stress load (DSL), external load monitoring, internal load monitoring, soccer competition performance, elite professional soccer

## Abstract

**Highlights:**

**What are the main findings?**

- Weekly dynamic stress load (DSL) volume was negatively associated with second-half high-speed running distance (HSRD) and sprint distance (SPRD), and with reduced full-match total distance covered (TDC), HSRD, SPRD, and high-intensity acceleration count, indicating that stronger associations were evident during the latter stages of match-play and across the full match.
- Weekly DSL volume was positively associated with time spent > 85% of heart rate maximum (HRmax) in both match halves, suggesting that higher weekly DSL volume was accompanied by greater exposure to very high cardiovascular strain during competition.

**What are the implications of the main findings?**

- Weekly DSL may serve as a practical microcycle load/fatigue indicator in this sample, particularly for monitoring high-intensity late-match performance capacity. However, these findings should be interpreted with caution and require confirmation in larger cohorts.
- The integration of weekly DSL volume with GPS-derived locomotor metrics and heart rate (HR)-based internal load measures may provide additional information to support load-management decisions by improving the contextualization of match running outputs and internal cardiovascular strain.

**Abstract:**

The aim of the current study was to examine whether weekly dynamic stress load (DSL) volume could be associated with competition internal and external load outcomes in professional soccer players. Weekly DSL volume was recorded across standardized one-match microcycles. Match outcomes included total distance covered (TDC), high-speed running distance (HSRD), sprint distance (SPRD), high-intensity accelerations (HIACC), high-intensity decelerations (HIDEC), high-metabolic-load distance (HMLD), time spent > 85% of maximum heart rate (HRmax), and Edwards training impulse (Edwards’ TRIMP). Analyses of our results revealed that higher weekly DSL volume was associated with greater time > 85%HRmax in the first half (β = 0.00647; *p* = 0.002) and second half (β = 0.00764; *p* = 0.026). In the second half, weekly DSL was negatively associated with HSRD (β = −0.3068; *p* < 0.001) and SPRD (β = −0.0619; *p* < 0.001), and positively with HMLD (β = 0.3532; *p* = 0.002). Across the full match, weekly DSL was negatively associated with TDC (β = −0.5080; *p* = 0.002), HSRD (β = −0.4159; *p* < 0.001), SPRD (β = −0.0988; *p* < 0.001), HIACC (β = −0.0265; *p* = 0.003), and Edwards’ TRIMP (β = −0.2251; *p* = 0.001). Weekly DSL volume may represent an important monitoring tool providing useful information for practitioners aiming to manage fatigue and support competition performance maintenance; however, these findings should be interpreted cautiously until confirmed in larger samples.

## 1. Introduction

In professional soccer, training load management has long been considered a core principle applied in practice for decades [1]. A plethora of scientific evidence has well demonstrated it to be fundamental for optimal competition performance and for minimizing players’ injury risk [2]. To date, its importance is further highlighted based on the observations revealing a progression in the soccer competition workload profile towards both higher intensity and volume [3]. This evolution in competitive game demands has further increased the need for detailed physical load assessment and monitoring during both training and competition, meeting the primary aim of soccer practitioners, which is decoding the game characteristics and understanding the effects of the loads players are exposed to. This training load monitoring, quantification, and analysis process has been common practice amongst soccer practitioners.

This methodological approach has been found to be vital for determining the conditioning needs of players in order to serve as a guide for the training process [4], assisting coaches’ decision-making process for the necessary training load modifications across the weekly microcycle in order to avoid an inappropriate stimulus. Since weekly training load distribution has been suggested to significantly influence competition performance, the assessment of training load, both internal and external, could establish a work ratio enhancing physical performance and promoting readiness to compete, while avoiding undesired overreaching, fatigue, and injury incidents [4,5]. To date, at the elite level of professional soccer, meeting the demands of an efficiently structured weekly microcycle and meeting the competition-conditioning demands have been achieved with the employment of global positioning system (GPS) devices [6,7]. The most commonly explored parameters on which training and competition load analyses have heavily relied involve certain GPS metrics, namely total distance (TDC), high-speed running distance (HSRD), sprint distance (SPRD) covered, and the high-intensity acceleration (HIACC) and deceleration (HIDEC) counts [8,9]. While those traditionally examined metrics have been widely accepted to provide valuable insights regarding the training load stress players have to cope with during training and/or competitions, they are not informative regarding the mechanical stress imposed on the players. Taking into consideration the nature of soccer as a sport, during which high-intensity mechanical actions are continuously required [10], recent scientific evidence indicates that accelerometer-derived external workload variables should be integrated into analyses alongside traditional external and internal load metrics, to provide additional insight into the mechanical demands and stresses imposed on the players during training and competitions [11].

The latest technological advancements in the integration of accelerometer technology in GPS units that feature triaxial accelerometers have provided the capacity for quantifying, apart from the traditional locomotor metrics, important impact metrics that are associated with contact team sports such as soccer [12]. One of those metrics, which has been relatively recently introduced but not extensively analyzed, is dynamic stress load (DSL). As a metric, DSL integrates triaxial accelerometer data to produce a weighted score representing cumulative dynamic mechanical stress, expressed in arbitrary units (AU) [12,13]. This metric (i.e., DSL) is intended to reflect the total body load impact over the whole training session by quantifying weighted impacts derived from triaxial accelerations (applying an impact threshold, e.g., >2 g), therefore representing cumulative mechanical stress exposure across the performed activities [12]. Notably, although a growing body of research has been devoted to other acceleration-based and mechanical metrics, this is not the case for DSL. The comparatively limited scientific literature, to the best knowledge of the authors, has aimed to examine its possible relationship with training load, and this provides inconsistent findings. From the available evidence, a number of studies have reported that DSL could serve as a valuable tool for training load monitoring. In particular, there are reports suggesting it is a potential indicator of both neuromuscular and mechanical fatigue [13,14] and is related to injury risk [15], while its levels have been found to be associated positively and negatively with internal and external load metrics (TDC, HSRD, and acceleration and deceleration distance), respectively [16]. Negative associations of DSL and internal load have also been reported in soccer [17,18,19], although these findings are not universal [20]. Negative associations between DSL and internal load (e.g., session rate of perceived exertion [sRPE]) have also been reported in soccer [21], although DSL may not consistently reflect fatigue-related changes across contexts [20].

Based on the available literature, although these findings come from a variety of sporting disciplines, including soccer, it could be suggested that DSL could serve as an effective metric in soccer, as it can provide useful information about players’ responses to training and/or competition-related stress. The importance of these suggestions is further increased when taking into consideration the possibility of a variable that could be less affected by contextual influences compared to traditional locomotor metrics, while still providing direct insight into mechanical loading [12,22,23]. Taking into consideration the possibility of the existence of a specific variable that could remain less affected by several contextual factors during training and/or competitions and be associated with both internal and external load metrics, while at the same time providing information related to fatigue and injury risk, increases the interest in examining this variable. In particular, it is of interest to examine whether it could provide information concerning training load and its association with readiness to compete and/or competition performance maintenance.

Therefore, the aim of the current study was to evaluate the possible association of weekly DSL with competition-derived internal and external load metrics, examining whether this variable could provide information regarding both training load periodization and competition fatigue kinetics. Our working hypothesis was that weekly DSL volume would be associated with the subsequent competition-derived internal and external load metrics and, in particular, time spent above 85% of individual maximal heart rate (time spent in minutes > 85% of HRmax), Edwards’ TRIMP, total distance covered (TDC (m)), HSRD (m), SPRD (m), and HIACC (>3 m·s^−2^) and HIDEC (<−3 m·s^−2^) count.

## 2. Materials and Methods

### 2.1. Study Design and Participants

The duration of this observational longitudinal study was that of a full soccer competition season. In order to examine the experimental questions, weekly microcycle DSL volume data were recorded in order to investigate their possible relationships with specific internal and external load metrics during the three different competition segments examined, namely the first half, the second half, and the full competition, in a cohort of elite professional male football players.

Regarding weekly microcycles, data collection for analyses throughout the study was employed only for those situated within a 1-game week, 6 days apart from the previous competition, and with the same structure. More specifically, during each one of these weeks, players participated in five (n = 5) training sessions, usually scheduled on the same days (i.e., match day [MD], plus [+], or minus [−]; MD + 2, MD − 4, MD − 3, MD − 2, and MD − 1), while MD + 1 was a day off, an approach that has been commonly employed in professional soccer [24].

In order for players to be included in this study, they had to fulfill the following criteria: (a) to be chosen as starters; (b) to have participated in the whole 1st half (full time and extra time) for the examination of the possible relationship with 1st-half competition external and internal load metrics and the weekly DSL volume; (c) to have participated in the whole 2nd half (full time and extra time) for the examination of the possible relationship with 2nd-half competition external and internal load metrics and the weekly DSL volume; (d) to have participated in the full competition (full time and extra time) for the examination of the possible relationship with the full competition external and internal load metrics and the weekly DSL volume; (e) to have participated in the full number of training sessions of the specific weekly microcycles included in this study (i.e., absence from any weekly session or sessions due to musculoskeletal and neurological pathologies such as muscle or tendon strain and joint or ligament sprains or overuse injury-related issues, or any other factors); (f) the players from each of the (a), (b), (c) and (d) criteria had to have participated in every single of the competitions examined, fulfilling criteria (a)–(d), in order to have exactly the same individuals for analyses during each one of the weekly microcycles included in the analyses for evaluating the associations of weekly DSL volume values with the obtained 1st-half, 2nd-half and full-competition external and internal load metrics. In addition, during the whole experimental period, all players were using the same GPS devices (i.e., the same unit for each player throughout the season). Therefore, the obtained DSL values that were selected for the analyses were compared only within the same player, ensuring the integrity and reliability of the data according to the manufacturer instructions [25], since each player has different biomechanics, injury history and fatigue levels, parameters that all play a significant role in determining how a player deals with the strain of each session [26,27] and in the generation of DSL.

Based on the aforementioned weekly microcycle and player selection criteria, from a total pool of twenty-four in-field professional male soccer players, who were members of a Greek Superleague team, ten (n = 10) players (mean age: 25.7 ± 5.14 years; height: 1.83 ± 0.07 m; body weight: 77.2 ± 8.68 kg, body fat %: 7.3 ± 1.41) during fifteen (n = 15) competitions were finally selected for examining the possible association of weekly DSL volume with the 1st-half internal and external metrics outcome. Regarding the selection criteria for assessing the possible relationships between weekly DSL volume and both the 2nd-half and the full-game internal and external load data, six (n = 6) of the aforementioned players (mean age: 26.5 ± 6.47 years; height: 1.80 ± 0.07 m; body weight: 75.9 ± 8.98 kg, body fat %: 6.5 ± 1.01) that participated in ten (n = 10) competitions fulfilled the respective criteria and were selected. It should be highlighted that the ten (n = 10) competitions that were employed in the analyses in this study for the evaluation of the association of weekly DSL volume and the examined competition load-derived metrics for the 2nd half and the full game were selected from the initial fifteen (n = 15) employed for the 1st-half analyses, in which the players participated in the full game. Notably, goalkeepers, due to their different training and competition load compared to in-field players, were excluded from the study.

All players provided written informed consent in accordance with the ethical guidelines of the Helsinki Declaration [28]. Data were obtained from the football club as the players were monitored daily, throughout the whole season, for training load monitoring purposes. Although ethics committee clearance, as is usually performed in research procedures, was not required [29], the study protocol was reviewed by the responsible institutional ethics committee, and ethical approval was granted (CREC 644/2025, 5 December 2025).

### 2.2. Procedures

#### 2.2.1. Anthropometry

Height was measured using a stadiometer (Charder HM210D, Charder Electronics Co., Ltd., Taichung City, Taiwan), weight was obtained using an electronic weight scale (Seca Alpha 770, Seca Vogel, Hamburg, Germany), and body-fat percentage was assessed by skinfold thickness measurement (Lange Skinfold Caliper, Cambridge Scientific Industries, Inc., Cambridge, MD, USA) using the 4-site formula proposed by Durnin and Womersley [30]. It should be mentioned that anthropometric characteristics of the study participants were assessed for descriptive purposes only and were not included in subsequent analyses.

#### 2.2.2. GPS Data Collection

Players’ outputs were collected by the STATSport GPS (Apex 10 Hz; 100 Hz tri-axial accelerometer, and 10 Hz magnetometer), a system that has been shown to be a valid and reliable assessment marker for monitoring team-sport movement demands [31]. STATSports metrics were processed using the manufacturer’s proprietary software Sonra (Version 2.0, STATSports, Newry, Northern Ireland, UK). For the purposes of the current study, the following external and internal load variables were measured: dynamic stress load (DSL; the weighted sum of acceleration events ≥ 2 g) [32,33], total distance covered in meters [TDC (m)], high-speed running distance in meters [HSRD (m); (>19.8 km/h)], sprint-running distance in meters [SPRD (m); (>25.5 km/h)], high-intensity acceleration number [HIACC (n); (>3 m/s^2^)], high-intensity deceleration number [HIDEC (n); (<−3 m/s^2^)], high-metabolic-load distance [HMLD (m)], time spent > 85% of individual maximum heart rate (minutes), and Edwards training impulse (Edwards’ TRIMP).

Dynamic stress load was calculated automatically using the custom algorithm included in the proprietary software provided by the manufacturer [14]. The internal load metrics time spent > 85% of HRmax and Edwards’ TRIMP were quantified using heart rate-derived measures collected with Polar Verity Sense optical sensors (Polar Electro Oy, Kempele, Finland), worn on the upper arm in accordance with manufacturer guidelines. Heart rate data were transmitted via Bluetooth and integrated with the STATSports APEX GPS, which is compatible with Bluetooth-enabled heart rate monitors. All heart rate-derived variables were subsequently calculated within the Sonra Version 2.0 (STATSports, Newry, Northern Ireland, UK) software environment. Time spent > 85% of HRmax was calculated for each official match and expressed in minutes. Edwards’ TRIMP was calculated using the same heart rate data, based on a zone-weighted method reflecting the duration and intensity of exercise [34]. All heart rate data were collected using Polar Verity Sense sensors and integrated within the STATSports GPS monitoring system for analysis. This metric’s values were obtained from the duration of the time spent in predefined heart rate zones relative to individual HRmax, with corresponding weighting factors applied to reflect the intensity of the performed exercise, providing an internal training load index synchronized with the recorded external load metrics. Notably, time spent > 85% HRmax and Edwards’ TRIMP were derived from the same heart rate signal. It should be mentioned that, due to the fact that both time spent > 85% HRmax and Edwards’ TRIMP are derived from the same heart rate signal, those variables are not actually fully independent. However, since Edwards’ TRIMP reflects a weighted accumulation of time across multiple heart rate zones, whilst time > 85% HRmax captures only the specific fragment above 85% of HRmax, although related, those two variables do represent distinct aspects of internal load and, therefore, should not be considered interchangeable.

During the whole experimental period, players were using the same GPS devices due to the following reasons: (i) in order to avoid interunit error and to maintain the integrity and reliability of the obtained data according to the manufacturer instructions [35] and (ii) based on recent reports regarding the employment of DSL suggesting to avoid interchanging GPS units between athletes and sessions in order to minimize the impact of poor interunit agreement [36].

#### 2.2.3. Maximum Heart Rate Assessment

Individual maximum heart rate (HRmax) was determined using heart rate monitors (Verity Sense optical sensors, Polar Electro Oy, Kempele, Finland) and obtained either during an incremental treadmill test until volitional exhaustion prior to the beginning of the season or any higher value during training and/or competition. The highest individual heart rate value recorded during the test was defined as HRmax.

#### 2.2.4. Statistical Analyses

Descriptive statistics are presented as mean ± standard deviation (SD). Associations between weekly dynamic stress load and match-derived internal and external load metrics were examined using linear mixed-effects models fitted in IBM SPSS Statistics (Version 21, IBM Corp., Armonk, NY, USA) with restricted maximum likelihood (REML). Weekly DSL was mean-centered (DSL_c) prior to analysis, and separate models were performed for first-half, second-half, and full-match datasets. In all models, competition-derived internal and external load variables were specified as dependent variables, while centered weekly DSL (DSL_c) was entered as the fixed predictor. Thus, the modeling approach reflects the temporal and physiological sequence whereby weekly DSL precedes and may influence subsequent competition-derived load metrics.

For each dependent variable (TDC, HSRD, SPRD as defined in the dataset, HIACC, HIDEC, HMLD, time > 85% HRmax, and Edwards’ TRIMP), DSL_c was entered as a fixed effect. Repeated observations across weeks were modeled with a first-order autoregressive residual covariance structure (AR(1)) for week_id within player_id. A random intercept for player_id was included when estimation was stable; when inclusion of the random intercept produced convergence/estimation instability (e.g., non-convergence, non-positive definite Hessian, or redundant/near-zero variance components), results were reported from the reduced model (fixed effect + AR(1) repeated structure). In cases where the random intercept for player_id was removed due to estimation instability, the corresponding variance component was estimated to be near-zero, indicating negligible between-player variability relative to within-player variability. Model assumptions were checked by visual inspection of residual diagnostics. Fixed effects were evaluated using Type III tests, and results are reported as unstandardized coefficients (β) with standard errors (SE), *t*-values, degrees of freedom, 95% confidence intervals, and *p*-values; statistical significance was set at *p* < 0.05. In our analyses, β represents the unstandardized regression coefficient, indicating the expected change in the dependent variable for a one-unit increase in centered weekly DSL (DSL_c), expressed in the original units of the respective outcome variable.

In addition to fixed-effect estimates, model performance was further evaluated using root mean square error (RMSE) and mean absolute error (MAE), calculated from model residuals, to provide an index of model error and complementary model-performance information. To further assess the stability of the principal findings under the limited sample size, leave-one-player-out sensitivity analyses were performed for the key significant models by repeating each analysis after excluding one player at a time.

## 3. Results

**Figure 1:**
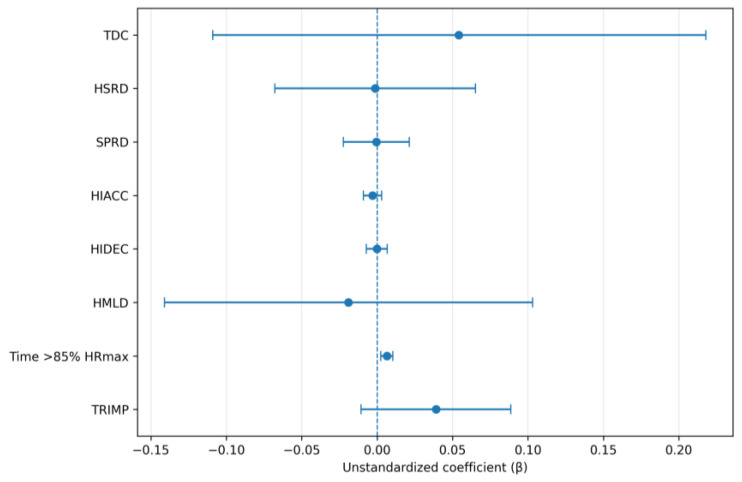
Coefficient plots for first-half linear mixed-effects models examining associations between weekly dynamic stress load and first-half match outcomes. Points represent fixed-effect estimates and horizontal bars represent 95% confidence intervals.

In the first half, weekly DSL was not associated with most match-derived outcomes. A significant positive association was observed only for time spent > 85% HRmax, indicating that higher weekly DSL was associated with greater first-half exposure to high cardiovascular strain (β = 0.00647, SE = 0.00201, 95% CI 0.00249 to 0.01045, *p* = 0.002). No significant associations were observed for TDC, HSRD, SPRD, HIACC, HIDEC, HMLD, or Edwards’ TRIMP (all *p* > 0.05; Table 1). These associations are illustrated in Figure 1.

**Figure 2:**
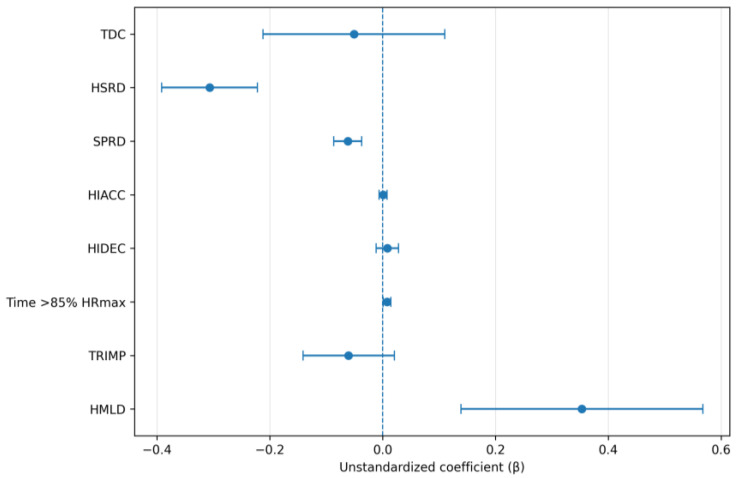
Coefficient plots for second-half linear mixed-effects models examining associations between weekly dynamic stress load and second-half match outcomes. Points represent fixed-effect estimates and horizontal bars represent 95% confidence intervals.

In the second half, weekly DSL showed significant negative associations with HSRD (β = −0.3068, SE = 0.0424, 95% CI −0.392 to −0.222, *p* < 0.001) and SPRD (β = −0.0619, SE = 0.0124, 95% CI −0.0866 to −0.0371, *p* < 0.001), and significant positive associations with HMLD (β = 0.3532, SE = 0.1070, 95% CI 0.1389 to 0.5676, *p* = 0.002) and time spent > 85% HRmax (β = 0.00764, SE = 0.00334, 95% CI 0.000944 to 0.01434, *p* = 0.026). No significant associations were identified for TDC, HIACC, HIDEC, or Edwards’ TRIMP (all *p* > 0.05; Table 2). The second-half model estimates are shown in Figure 2.

**Figure 3:**
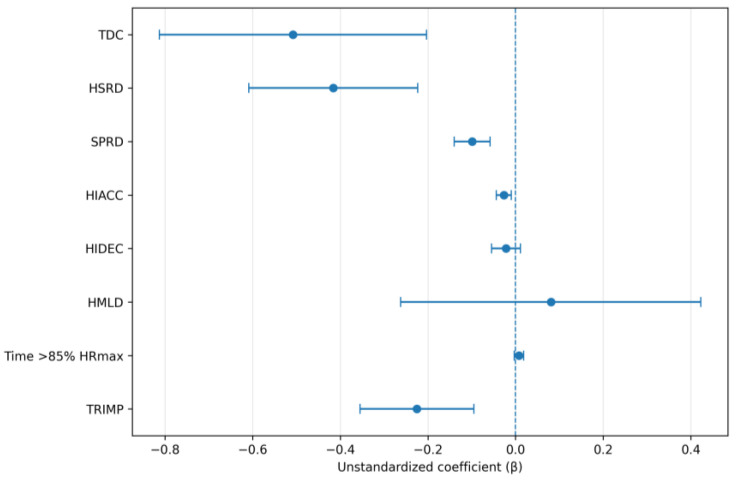
Coefficient plots for full-competition linear mixed-effects models examining associations between weekly dynamic stress load and full-competition match outcomes. Points represent fixed-effect estimates and horizontal bars represent 95% confidence intervals.

Across the full competition, higher weekly DSL was associated with lower TDC (β = −0.5080, SE = 0.1519, 95% CI −0.813 to −0.203, *p* = 0.002), lower HSRD (β = −0.4159, SE = 0.0967, 95% CI −0.609 to −0.223, *p* < 0.001), lower SPRD (β = −0.0988, SE = 0.0205, 95% CI −0.140 to −0.0581, *p* < 0.001), lower HIACC (β = −0.0265, SE = 0.00867, 95% CI −0.0437 to −0.00937, *p* = 0.004), and lower Edwards’ TRIMP (β = −0.2251, SE = 0.0650, 95% CI −0.355 to −0.0950, *p* = 0.001). No significant associations were observed for HIDEC, HMLD, or time spent > 85% HRmax (all *p* > 0.05; Table 3). The full-competition model estimates are presented in Figure 3.

In addition to the fixed-effect estimates for each analysis, RMSE and MAE values are reported in Table 1, Table 2 and Table 3 to provide complementary information on model error. To further evaluate the robustness of the main findings under the limited sample size, leave-one-player-out sensitivity analyses were performed for the key significant models. In the first half, the positive association between weekly DSL and time spent > 85% HRmax remained directionally consistent and statistically significant across reruns. In the second half, the associations with HSRD, SPRD, and HMLD remained statistically significant across reruns, whereas the association with time spent > 85% HRmax remained positive but was not statistically significant in every omission. Across the full competition, the negative associations with HSRD, SPRD, HIACC, and Edwards’ TRIMP remained statistically significant across reruns, whereas the association with TDC showed greater sensitivity to player omission and should therefore be interpreted more cautiously. Overall, these sensitivity analyses supported the directional robustness of the principal findings, although some associations appeared more sensitive to player omission and should be interpreted cautiously.

## 4. Discussion

The aim of the current study was to examine the associations between weekly DSL volume and competition-derived external and internal load metrics in professional soccer players. In particular, the objective was to determine whether weekly DSL volume was associated with official competition outcomes (TDC, HSRD, SPRD, HIACC and HIDEC count, HMLD, and time > 85% HRmax), with competition responses evaluated for the first half, the second half, and the full match. In agreement with our hypothesis, analyses revealed that weekly DSL volume was significantly associated with several second-half and full-competition outcomes, whereas associations in the first half were limited to internal load. Specifically, higher weekly DSL volume was related to lower second-half HSRD and SPRD, and lower full-match TDC, HSRD, SPRD, and HIACC count. Notably, although not significant, a trend of a positive relationship between weekly DSL volume and time spent > 85% HRmax was observed, indicating a tendency for higher weekly DSL to be accompanied by greater cardiovascular strain.

The observed positive relationship in the first half between weekly DSL volume and time spent > 85% HRmax is comparable with evidence reporting a strong association between DSL and internal load in soccer [13,33]. Notably, although none of the aforementioned studies examined the association of DSL with time spent > 85% HRmax, but rather with rating of perceived exertion (RPE), their findings provide indirect support for our observations. This is supported by the fact that RPE has been found to be significantly associated with HR-based internal load measures [37]. A possible mechanism underpinning this finding could be based on the evidence indicating that DSL could serve as a potential indicator of both neuromuscular and mechanical fatigue [13,14]. According to these reports, higher weekly DSL volume could result in increased neuromuscular residual fatigue and induced muscle damage, factors that have been found to negatively affect readiness to compete via motor impairment [38,39]. These training-induced effects have been suggested to increase physiological strain and the effort required to maintain performance levels [40]. Specifically, accumulated stress-induced neuromuscular fatigue has been reported to reduce movement economy and elevate cardiovascular strain for a given external output [40], whilst muscle damage can alter recruitment patterns and coordination, and increase the cost of producing the same running profile (e.g., greater co-contraction and stiffness strategies), factors that could result in elevated HR responses [38]. In this case, competing under a state of fatigue would likely increase internal load, since players are required to exert greater physiological strain in order to cope with the competition demands [13]. Therefore, competing under a state of fatigue would likely increase internal load by resulting in elevated physiological strain in order to cope with the competition demands, a suggestion that is in line with our findings.

The absence of an observed effect of weekly DSL volume on the other external load metrics obtained during the first half of the competition could be partly explained by observations that, even though increases in physiological strain and/or performing with accumulated fatigue could be evident, external load outcomes, including locomotor outputs, could remain unaffected [4,41]. More specifically, in order to cope with the specific actions encountered within competition, the expected external load output could be maintained at the desired level but with higher physiological stress and perception of effort due to the fatigue state in which players entered the competition [4]. In addition, another plausible explanation for the lack of an observed association between weekly DSL volume and the external load metrics could be related to the well-demonstrated finding that, although players may compete under fatigue, decreases in locomotor activities and intensive actions mostly occur towards the end of the competition. Indeed, in soccer, fatigue expressed by reduced external load outputs has been mostly observed in the second half and, more specifically, towards the end of the competition [20,40,41,42,43,44], rather than during the first half. It should be mentioned that this absence of an effect of weekly DSL volume on external competition metrics is also plausible, since internal strain can increase even when locomotor output does not, as a result of other physiological responses resulting in cardiovascular drift (i.e., progressive HR rise at a constant workload), such as thermoregulatory demands and hydration status, which could accentuate this internal–external differential response pattern [41,45,46,47]. Regarding the other internal load metric, competition-derived Edwards’ TRIMP, the absence of an observed association with weekly DSL volume could be explained by the composite nature of this measure. Unlike time spent > 85% HRmax, Edwards’ TRIMP is not solely dependent on HR responses [4]. This internal load measure reflects accumulated time across different HR zones and is highly influenced by locomotor volume [4]. Since no associations were evident between weekly DSL volume and competition locomotor outputs, and given that time spent > 85% HRmax corresponds only to the upper Edwards’ TRIMP categories (i.e., 80–90% and 90–100% of HRmax), any increase in cardiovascular strain at higher intensities in our study may not have been sufficient to affect the overall Edwards’ TRIMP score. Indeed, Edwards’ TRIMP reflects the whole HR-intensity distribution during match-play, and any modest increase in time spent in the high-HR zones could have been attenuated by time accumulated in the lower-intensity zones, resulting in stability of the overall weighted sum.

In contrast to the first half, the second-half analyses revealed several relationships between weekly DSL volume and the examined competition-derived internal and external load metrics. The external-load-metric analyses revealed significant negative associations between weekly DSL volume and second-half competition-derived HSRD and SPRD, whilst a positive association was observed with HMLD. Our findings regarding HSRD and SPRD are comparable with the limited evidence indicating that DSL magnitude can be used as a fatigue indicator [13]. In this context, observed increases in weekly DSL volume could be indicative of greater residual and mechanical fatigue, which could in turn be reflected by reduced capacity to perform adequately during competition. In support of our findings, this effect could be more evident during the second half of competitions, a suggestion supported by the large body of literature demonstrating performance deterioration towards the end of the game [20,40,42,43,44]. In other words, higher weekly DSL could be accompanied by impaired readiness to compete, which could be more evident during the second half, providing justification for our observations. This association between weekly DSL volume and the inverse response of HSRD and SPRD highlights the importance of weekly DSL as an indicator of fatigue, supporting the limited evidence on this topic [13]. The mechanisms underpinning reductions in HSRD and TDC in the presence of higher weekly DSL volume are well-described in the literature and include impaired force control, training-induced muscle damage, and residual neuromuscular fatigue [38,39,48]. Since higher weekly DSL implies higher levels of cumulative mechanical stress across the week, this could be accompanied by players competing in a fatigued state.

Regarding the observed positive relationship between weekly DSL and HMLD in our second-half analysis, the available literature providing direct association analyses is extremely limited. Thus, the interpretation of our findings is based on the definition of HMLD and the types of actions contributing to it. More specifically, HMLD represents distance covered when estimated metabolic power exceeds >25.5 W·kg^−1^, and it includes not only high-speed running but also acceleration and deceleration bouts [49]. In parallel, DSL is an accelerometer-derived indicator reflecting accumulated weighted high-magnitude impacts (commonly using thresholds > 2 g) and is therefore driven by high-impact locomotor actions and repeated changes in velocity [13,50]. Thus, when high-speed efforts are reduced, a higher contribution of intermittent acceleration–deceleration activity could still support elevated HMLD, since the metabolic-power classification also captures these non-high-speed, high-cost actions.

Interestingly, in contrast to our hypotheses, no significant relationship was evident between weekly DSL volume and second-half-derived high-intensity acceleration and deceleration count. DSL has been suggested to reflect cumulative neuromuscular and mechanical stress from accelerations, decelerations, changes in direction, and high-impact actions [13,51]. These components have been linked to neuromuscular fatigue, eccentric muscle damage, and reductions in force production ability [52,53], whilst high-intensity accelerations and decelerations have been reported to be among the most fatigue-sensitive external load outputs [51]. Therefore, based on evidence indicating that high-intensity accelerations and decelerations are reduced in later stages of soccer competitions, coinciding with markers of fatigue [52], and that they can also be negatively affected in competition by increased weekly training load [54], a negative relationship was expected between weekly accumulated DSL volume and competition-obtained high-intensity acceleration and deceleration count in our study. However, this absence of an effect may be attributable to several plausible factors. Firstly, count-based acceleration and deceleration measures using fixed thresholds (e.g., ±3 m·s^−2^) may not adequately capture fatigue-related decrements in movement intensity, especially if players still exceed this threshold under fatigue, while larger-magnitude actions (e.g., >4 m·s^−2^) could be more sensitive to neuromuscular impairment. Secondly, even when acceleration and deceleration activity is altered, distance-based metrics could be more sensitive than counts, since the latter provide limited information regarding the magnitude and duration of exposure to high mechanical demands [15]. Lastly, these metrics have been reported to be strongly affected by contextual factors [51], and acceleration/deceleration profiles have been found to vary significantly due to the variabilities in match context and tactical constraints [55].

Regarding the two internal load metrics, our findings revealed that weekly DSL was positively associated with time spent > 85% HRmax during the second half, whereas no significant association was observed with Edwards’ TRIMP. This is consistent with the first-half observation, indicating that the higher the weekly DSL volume, the higher the cardiovascular strain players faced during the second half of competition. This finding, in conjunction with the first-half observation, supports the suggestion of weekly DSL values serving as sensitive indicators of accumulated fatigue [13]. The mechanisms underpinning this finding most probably involve residual neuromuscular and mechanical fatigue and impaired power output due to increased weekly DSL volume, resulting in elevated cardiovascular demands for a given external output [38,48]. Regarding Edwards’ TRIMP, the lack of an association was most probably related to its composite nature and to a compensatory effect of increased time spent in lower-intensity activity despite reductions in high-speed actions. Notably, HR-related strain increases during the second half could also be related to factors other than fatigue, such as thermoregulatory strain and dehydration [41,47]. This pattern is in agreement with our first-half findings, where higher weekly DSL volume was likewise linked to greater exposure to very high cardiovascular strain (i.e., time in the “red zone”), suggesting a consistent relationship between accumulated weekly mechanical load and high-intensity internal responses across match halves.

The full-match analyses revealed negative associations of several external load metrics with weekly DSL volume. In particular, in accordance with our second-half observations, a significant negative association between weekly DSL volume and HSRD and SPRD was observed. In addition, for the first time in our study, an inverse association was evident between weekly DSL volume and TDC and HIACC count. Although no studies have examined the effects of accumulated weekly DSL volume on key full-competition-derived external load indicators in soccer, these observations are comparable with the available literature regarding TDC and HSRD [13,15], and HIACC-related outputs in team-sport contexts [15]. Indeed, higher DSL volumes have previously been reported to be negatively associated with TDC and HSRD [13,15]. Indirect support for this pattern also comes from evidence showing inverse relationships between weekly training load and competition external load outcomes, considering that accumulated DSL volume reflects higher weekly mechanical load and, therefore, increased fatigue levels; specifically, higher weekly load has been associated with reductions in match-play TDC, HSRD, and SPRD [56]. In addition, negative associations of DSL levels with distance-, rather than count-based, acceleration outputs have also been reported [18]. Specifically, for TDC, its observed negative association with weekly DSL volume during the full match is indirectly supported by evidence showing that increased weekly load negatively affects this external load metric [56]. This relationship could be discussed in the context of evidence indicating that entering competition in a training-load-induced fatigued state has been associated with reduced intensive locomotor actions such as HSRD, SPRD, and high-intensity acceleration output [38,39,51]. In addition, the lack of an association between weekly DSL and first- and second-half TDC, compared with the whole-match values, could be partly attributable to greater contextual variability within halves compared with the whole match, which could mask possible fatigue-related effects on total distance within shorter time windows [57]. The findings in our full-match analyses regarding HSRD, SPRD, and HIACC could be supported by the suggestion of weekly DSL volume being a fatigue indicator [13]. In particular, increased weekly DSL load could be linked with neuromuscular and mechanical fatigue, since it has been proposed to reflect the accumulation of weighted high-magnitude impacts > 2 g resulting from intensive accelerations, decelerations, changes in direction, impacts, landings, and contacts [13,50]. These parameters have been repeatedly linked to increased residual neuromuscular fatigue and muscle damage, reduced force production and rate of force development, and a decline in the ability to repeatedly perform high-speed actions [38,48,51,58]. The findings regarding HSRD and SPRD are further supported by our second-half observations where similar associations were evident, indicating a decline in the capacity to produce high-intensity actions.

For the first time in our study, we observed a negative association with full-match HIACC count, a finding that is in agreement with our hypothesis. This negative association could most probably reflect accumulated neuromuscular and mechanical fatigue in relation to weekly DSL volume, considering that high-intensity accelerations have been reported to be among the most fatigue-sensitive external load metrics [51]. HIACC has also been reported to be strongly dependent on high rates of force development and neuromuscular function, capacities that could be negatively affected by accumulated mechanical stress [59,60]. The fact that this effect was only evident in the full-match data, and not during the first or second halves, could be explained by the shorter time window and fewer high-intensity events within each half, and thus higher random variability compared with the whole match [57]. Therefore, half-match data could fail to detect an association due to fewer acceleration actions and higher contextual variability, whilst the full match could more efficiently capture the cumulative reduction in this explosive type of action.

The lack of an association between weekly DSL and full-match HIDEC, although in contrast to our hypothesis, is consistent with the half-specific analyses. In addition, HIDEC has been suggested to be strongly dependent on contextual and tactical constraints, which could dominate variance and weaken any load-limited association when treating them as purely fatigue-associated outputs [51,55]. From a biomechanical standpoint, this absence of an observed association is also plausible when contrasted with acceleration-based outputs, since accelerations are limited mainly by propulsive concentric force and rapid force production, whilst decelerations are characterized by greater braking load and a strong eccentric component needed to absorb rather than produce force [51,61]. Collectively, these factors provide a plausible explanation for the observed weekly DSL volume associations with acceleration- and speed-related outputs, but not with HIDEC [51].

Despite our hypotheses, no associations were evident between weekly DSL volume and HMLD in the full-match analyses. As previously reported, HMLD provides global information about total high-intensity activity, as it includes both high-velocity running and acceleration/deceleration contributions [49,62]. Therefore, the absence of an association could reflect a composite effect of reductions in HSRD/SPRD alongside the distinct contributions of acceleration and deceleration demands, in conjunction with contextual factors and the larger between-match variability of metabolically derived estimates [51,63,64], whilst the most demanding passages of play may not be fully represented when values are aggregated across the full 90 min duration [65].

Regarding internal load, our outcomes revealed that although weekly DSL volume was positively associated with time > 85% HRmax in both halves, the full-match analyses showed only a non-significant positive directional trend. A possible rationale supporting this finding could be that the expected fatigue-related increase in physiological strain—resulting from accumulated weekly DSL—could have been partly counterbalanced by the observed negative associations of the most physiologically demanding external actions (TDC, HSRD, SPRD, and HIACC) with weekly DSL volume. Lastly, Edwards’ TRIMP was inversely associated with weekly DSL volume, in contrast to the unaffected time spent > 85% HRmax, a pattern that is coherent with the composite nature of Edwards’ TRIMP being influenced by time accumulated across all HR intensities and thus being sensitive to a whole-match down-regulation of external output across 90 min.

## 5. Limitations

This study is subject to several limitations. One limitation of the present study is that DSL calculation is derived from a proprietary STATSports algorithm with a non-public weighting function, a parameter that limits both the transparency and reproducibility of the findings. Another important limitation is the relatively small number of players that were available for analysis, particularly in the second-half and full-match datasets. Although the repeated observations increased the number of data points and leave-one-player-out sensitivity analyses supported the directional robustness of the main findings of our study, some associations were sensitive to player omission and should therefore be interpreted cautiously until confirmation comes from studies with larger cohorts. Lastly, it should also be considered that Edwards’ TRIMP and time spent > 85% HRmax could partly overlap, since time spent at higher HR intensities is a contributor to Edwards’ TRIMP calculation. However, this contribution is not standard and could significantly vary according to the distribution of time spent across HR zones and competition-specific contextual demands; therefore, these two metrics are not equivalent and should be interpreted accordingly.

## 6. Conclusions

In conclusion, our findings indicate, in support of our hypothesis, that weekly DSL volume could serve as a valuable metric for weekly training load monitoring in relation to subsequent competition responses. In particular, weekly DSL volume was related to competition-derived internal load, with the most consistent associations observed for time spent > 85% HRmax during both match halves (first half and second half), indicating that higher weekly DSL volume was accompanied by greater cardiovascular strain during match-play. Regarding external load, the present data indicate that weekly DSL volume was more strongly related to high-intensity competition outputs and locomotor volume when these were evaluated during the latter stages of competition and across the full match, rather than during the first half. Specifically, weekly DSL volume was negatively associated with second-half HSRD and SPRD, and with full-competition TDC, HSRD, SPRD, and HIACC count, whilst first-half external load outputs were not consistently related to weekly DSL volume. Taken together, these findings support the rationale that weekly DSL monitoring could be particularly informative regarding late-match competition performance, where fatigue-related reductions in high-intensity running, sprinting, and locomotor output are more likely to become evident. However, it should be mentioned that these findings should be interpreted with caution and require replication in larger cohorts.

## 7. Practical Applications

The present findings suggest that weekly DSL volume could offer additional contextual information regarding accumulated weekly training load exposure and its potential implications for subsequent competition performance in this specific sample. In this context, weekly DSL volume could serve as an additional tool for coaches making load-management decisions aimed at monitoring fatigue and maintaining competition load outputs, particularly during the latter stages of the game. Specifically, practitioners, while considering both the findings and the limitations of this study, could consider elevated weekly DSL volume as being associated with (i) reduced HSRD and sprint-related output during the second half, (ii) reduced whole-match running outputs such as TDC and HIACC, and (iii) increased exposure to very high cardiovascular strain, defined as time spent above 85% HRmax. Notably, weekly DSL should be evaluated in conjunction with GPS-derived locomotor metrics and HR based internal load indices. These observations should be interpreted with caution and regarded as preliminary until other studies with the employment of larger cohorts provide further confirmation.

## Figures and Tables

**Table 1:** First-half linear mixed-effects models examining associations between weekly dynamic stress load (DSL_c) and first-half match outcomes.

Outcome	β	SE	df	*t*	*p*	95% CI	RMSE	MAE
TDC (m)	0.0541	0.0825	112.136	0.656	0.513	−0.109 to 0.218	397 m	316 m
HSRD (m)	−0.00125	0.0336	109.227	−0.037	0.970	−0.0678 to 0.0653	176 m	141 m
SPRD (m)	−0.000495	0.0110	92.066	−0.045	0.964	−0.0223 to 0.0213	56.6 m	44.9 m
HIACC (count)	−0.00307	0.00305	103.645	−1.006	0.317	−0.00913 to 0.00299	15.7	11.3
HIDEC (count)	−0.000201	0.00348	99.017	−0.058	0.954	−0.00711 to 0.00671	17.3	11.8
HMLD (m)	−0.0190	0.0613	103.586	−0.310	0.757	−0.141 to 0.103	334.4 m	248.1 m
Time > 85% HRmax (min)	0.00647	0.00201	90.364	3.226	0.002	0.00249 to 0.01045	10.7	8.7
Edwards’ TRIMP (AU)	0.0390	0.0250	94.729	1.561	0.122	−0.0106 to 0.0887	127.0	103.0

Abbreviations: β, unstandardized fixed-effect estimate for DSL_c; SE, standard error; CI, confidence interval; RMSE, root mean square error; MAE, mean absolute error; TDC, total distance covered; HSRD, high-speed running distance; SPRD, sprint distance; HIACC, high-intensity accelerations; HIDEC, high-intensity decelerations; HMLD, high-metabolic-load distance; HRmax, maximal heart rate; AU, arbitrary units.

**Table 2:** Second-half linear mixed-effects models examining associations between weekly dynamic stress load (DSL_c) and second-half match outcomes.

Outcome	β	SE	df	*t*	*p*	95% CI	RMSE	MAE
TDC (m)	−0.0508	0.0800	44.822	−0.635	0.528	−0.212 to 0.110	289 m	230 m
HSRD (m)	−0.3068	0.0424	51.631	−7.230	<0.001	−0.392 to −0.222	160 m	120 m
SPRD (m)	−0.0619	0.0124	57.263	−5.009	<0.001	−0.0866 to −0.0371	35 m	22 m
HIACC (count)	0.000753	0.00344	45.653	0.219	0.827	−0.00617 to 0.00767	11.2	8.5
HIDEC (count)	0.008543	0.00985	44.252	0.867	0.390	−0.0113 to 0.0284	30	17
HMLD (m)	0.3532	0.1070	55.870	3.302	0.002	0.1389 to 0.5676	300 m	230 m
Time > 85% HRmax (min)	0.00764	0.00334	55.962	2.286	0.026	0.000944 to 0.01434	8	6
Edwards’ TRIMP (AU)	−0.0604	0.0404	50.009	−1.498	0.140	−0.141 to 0.0206	115	85

Abbreviations: β, unstandardized fixed-effect estimate for DSL_c; SE, standard error; CI, confidence interval; RMSE, root mean square error; MAE, mean absolute error; TDC, total distance covered; HSRD, high-speed running distance; SPRD, sprint distance; HIACC, high-intensity accelerations; HIDEC, high-intensity decelerations; HMLD, high-metabolic-load distance; HRmax, maximal heart rate; AU, arbitrary units. HMLD was retained as a reduced repeated-only AR(1) model where the random intercept was redundant/unstable.

**Table 3:** Full-competition linear mixed-effects models examining associations between weekly dynamic stress load (DSL_c) and full-competition match outcomes.

Outcome	β	SE	df	*t*	*p*	95% CI	RMSE	MAE
TDC (m)	−0.5080	0.1519	50.014	−3.345	0.002	−0.813 to −0.203	3020 m	2580 m
HSRD (m)	−0.4159	0.0967	50.630	−4.301	<0.001	−0.609 to −0.223	300 m	240 m
SPRD (m)	−0.0988	0.0205	50.151	−4.825	<0.001	−0.140 to −0.0581	70 m	55 m
HIACC (count)	−0.0265	0.00867	48.218	−3.053	0.004	−0.0437 to −0.00937	45	32
HIDEC (count)	−0.0220	0.0166	46.953	−1.326	0.191	−0.0549 to 0.0110	45	32
HMLD (m)	0.0807	0.1720	50.486	0.469	0.641	−0.262 to 0.423	900 m	700 m
Time > 85% HRmax (min)	0.00789	0.00544	48.836	1.451	0.153	−0.00296 to 0.0187	15	11
Edwards’ TRIMP (AU)	−0.2251	0.0650	50.630	−3.473	0.001	−0.355 to −0.0950	230	170

Abbreviations: β, unstandardized fixed-effect estimate for DSL_c; SE, standard error; CI, confidence interval; RMSE, root mean square error; MAE, mean absolute error; TDC, total distance covered; HSRD, high-speed running distance; SPRD, sprint distance; HIACC, high-intensity accelerations; HIDEC, high-intensity decelerations; HMLD, high-metabolic-load distance; HRmax, maximal heart rate; AU, arbitrary units. For full competition, TDC retained a player-level random intercept; the remaining outcomes were fitted using repeated-only AR(1) structures.

## Data Availability

Data are available upon request to the corresponding author.

## Abbreviations

The following abbreviations are used in this manuscript:

DSL	Dynamic Stress Load
TDC	Total Distance Covered
HSRD	High-Speed Running Distance
SPRD	Sprint Distance
HIACC	High-Intensity Acceleration count (High-Intensity Accelerations)
HIDEC	High-Intensity Deceleration count (High-Intensity Decelerations)
HMLD	High-Metabolic-Load Distance
HR	Heart Rate
HRmax	Maximum Heart Rate
sRPE	session Rating of Perceived Exertion
TRIMP	Training Impulse
GPS	Global Positioning System
g	Gravitational acceleration (g-force; accelerometer threshold unit)
W·kg^−1^	Watts per kilogram (metabolic power unit)
m·s^−2^	Meters per second squared (acceleration/deceleration unit)
